# SCaBP3/CBL7 negatively regulates the plasma membrane H^+^-ATPase and modulates hypocotyl elongation in *Arabidopsis*

**DOI:** 10.1080/15592324.2022.2092699

**Published:** 2022-06-28

**Authors:** Xiao Liu, Yujiao Wu, Haiqi Fu, Shu Song, Qian He, Yongqing Yang

**Affiliations:** College of Biological Sciences, China Agricultural University, Beijing Haidian, China

**Keywords:** *Arabidopsis thaliana*, PM H^+^-ATPase, calcium binding protein SCaBP3, hypocotyl elongation

## Abstract

The regulation of hypocotyl elongation is an important process in plant growth and development and depends on the activity of the plasma membrane (PM) H^+^-ATPase. In this study, we found that the Arabidopsis protein SOS3-LIKE CALCIUM BINDING PROTEIN3 (SCaBP3) negatively regulates PM H^+^-ATPase activity in yeast and hypocotyl elongation in dark-grown seedlings. Yeast two-hybrid assays showed that SCaBP3 interacts with representative members of the Arabidopsis PM H^+^-ATPase family. Experiments in RS-72 yeast showed that SCaBP3 negatively regulates PM H^+^-ATPase activity-dependent yeast cell growth. Hypocotyl elongation was promoted in the loss-of-function mutant *scabp3* and inhibited in *SCaBP3* overexpression lines of Arabidopsis. We propose that SCaBP3 modulates hypocotyl elongation by negatively regulating PM H^+^-ATPase activity.

First proposed in the early 1970s, the acid growth hypothesis states that auxin-mediated plant cell growth depends on extracellular acidification caused by H^+^ efflux.^1–4^ During this process, the plasma membrane (PM) H^+^-ATPase hydrolyzes ATP to provide energy for the H^+^ efflux that acidifies the cell wall.^5–7^ Takahashi et al. demonstrated that auxin-mediated regulation of PM H^+^-ATPase activity could effectively regulate hypocotyl elongation.^8^ Studies have shown that SMALL AUXIN UP-RNA (SAUR) promotes the growth of Arabidopsis hypocotyl cells by interacting with the 2C protein phosphatase PP2C-D to upregulate PM H^+^-ATPase activity.^9^ In addition, the receptor kinase PEPTIDE CONTAINING SULFATED TYROSINE (PSY1), also interacts with PM H^+^-ATPase to participate in the regulation of hypocotyl elongation.^10^ In summary, the regulation of PM H^+^-ATPase activity seems to play an important role in modulating hypocotyl elongation.

In our previous study, SOS3-LIKE CALCIUM BINDING PROTEIN3 (SCaBP3) was found to interact with the C terminus of the PM H^+^-ATPase AHA2 and to inhibit AHA2 activity.^11^ To investigate potential interactions between SCaBP3 and other AHAs, we performed yeast two-hybrid assays. We first constructed a phylogenetic tree by software MEGA (Figure 1a) to analyze the relationships among the AHA family proteins. We then selected four representative AHAs for further study: the closest relative of AHA2, AHA1; the second closest relative, AHA3; a more distantly related protein, AHA9; and the most distant relative, AHA10. For the yeast two-hybrid assays, the C termini of AHA1, AHA3, AHA9, and AHA10 were cloned into the BD (*pGBKT7*) yeast expression vector. The full-length SCaBP3 coding region was cloned into the pGADT7 vector. Yeast strain AH109 was cotransformed with SCaBP3 and the C terminus of each of the AHAs or the empty *pGBKT7* vector. It was reported in previous study that PKS5 can interact with AHA2,^11^ and we used the interaction experiment of PKS5 and AHA2C as a positive control (Figure 1b). (Figure 1b). These experiments showed that SCaBP3 interacts with all AHAs tested, suggesting that SCaBP3 can act as a general regulator of PM H^+^-ATPases (Figure 1c, d).

**Figure 1.**
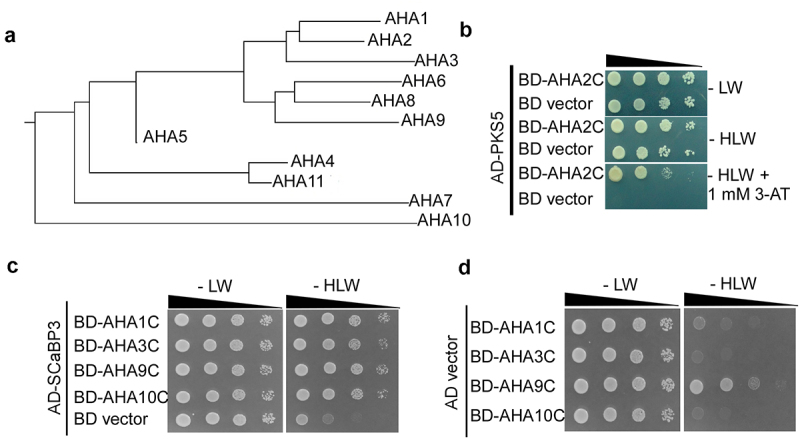
Interaction of SCaBP3 with AHA family members. (a). Evolutionary tree of the PM H^+^-ATPase family members of *Arabidopsis thaliana* by software MEGA. (b) Yeast two-hybrid assay of the interaction of PKS5 with AHA2C, as a positive control. (c) Yeast two-hybrid assay to analyze the interaction of SCaBP3 with AHA1C, AHA3C, AHA9C, AHA10C. (d) The negative control for the interaction of SCaBP3 with AHA1C, AHA3C, AHA9C, and AHA10C. For (b) and (c), yeast cells were observed and photographed after 3–5 days of growth on the corresponding medium.

Because SCaBP3 interacts with the C terminus of AHA2,^11^ we hypothesized that this interaction is critical for the negative regulation of PM H^+^-ATPase activity by SCaBP3. The introduction of SCaBP3 inhibited the growth of transgenic yeast RS-72 carrying pMP1745-AHA2 (Figure 2a, d). The vector pMP1745-AHA2Δ92 was constructed to express a truncated AHA2 that lacks the C-terminal 92 amino acids and is constitutively active.^12^ The expression of *SCaBP3* in the aha2Δ92 yeast strain had little effect on its growth, indicating that SCaBP3 did not affect the activity of aha2Δ92 (Figure 2b, d). However, SCaBP3 did inhibit the growth of transgenic yeast RS-72 carrying pMP1745-AHA2^P68S^ (an enhanced activity mutant of AHA2)^13^ (Figure 2c, d). We conclude that SCaBP3 inhibits PM H^+^-ATPase activity by directly interacting with the C terminus of AHA2 in yeast.

**Figure 2.**
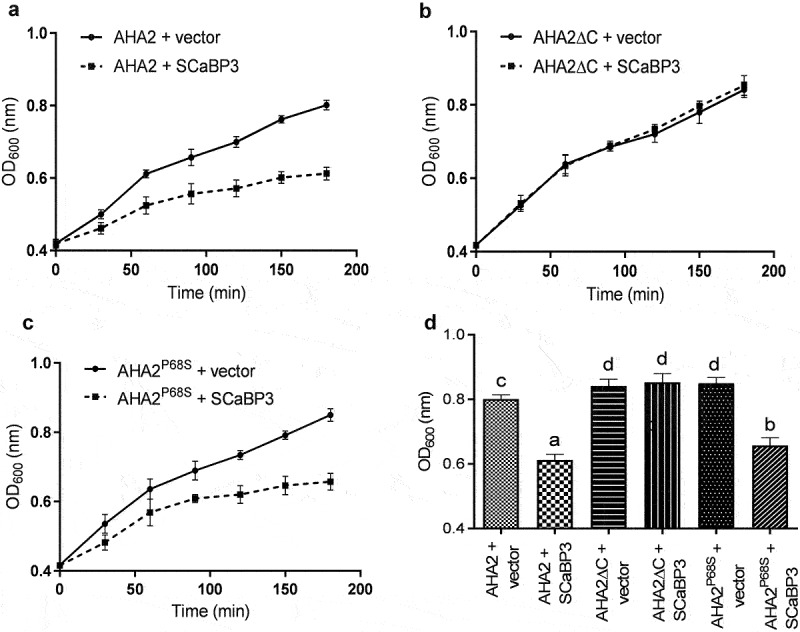
The effect of SCaBP3 on PM H^+^-ATPase activity in RS-72 yeast. (a) 1645-SCaBP3 or 1645 empty vector was co-expressed with 1745-AHA2 full-length protein in yeast cells (RS-72). The samples were shaken at 28°C (220 rpm/min), and yeast growth (OD_600_) was measured every 30 min for 3 hours using a microplate reader. All data represent means ± standard deviations (SD) of at least five replicates.(b) 1645-SCaBP3 or 1645 empty vector was co-expressed with a truncated 1745-AHA2 protein lacking the C terminus (AHA2Δ92 and AHA2 Δ C) in yeast cells (RS-72). The samples were shaken at 28°C (220 rpm/min), and yeast growth (OD_600_) was measured every 30 min for 3 hours using a microplate reader. All data represent means ± SD of at least five replicates. (c) 1645-SCaBP3 or 1645 empty vector was co-expressed with a mutated 1745-AHA2 protein (AHA2^P68S^) in yeast cells (RS-72). The samples were shaken at 28°C (220 rpm/min), and yeast growth (OD_600_) was measured every 30 min for 3 hours using a microplate reader. All data represent means ± SD of at least five replicates. (d) Comparison of PM H^+^-ATPase-dependent growth of RS-72 yeast at 180 min. All data represent means values ± SD of at least five replicate experiments. A one-way ANOVA was used to determine statistical significance; significant differences (P ≤ .05) are indicated by different lowercase letters.

This study demonstrates that SCaBP3 is a negative regulator of PM H^+^-ATPase activity and interacts with the C terminus of multiple members of the AHA family. Therefore, we hypothesized that the negative regulation of PM H^+^-ATPase activity by SCaBP3 may be involved in the regulation of diverse physiological processes during plant growth. To test this hypothesis, we investigated whether SCaBP3 is involved in the regulation of hypocotyl elongation. Seeds of Arabidopsis Col-0 (the wild type), the null mutant *scabp3* and its two complementation lines (COM 1–3 and COM 8–7), and two *SCaBP3* overexpression lines (OE-1 and OE-2) were sown on MS medium. The experimental group was germinated in the dark (dishes wrapped and kept in the dark). The controls were incubated in continuous white light. After 4 days of growth, we measured the seedling hypocotyl lengths. Under white light conditions, the hypocotyls of the *scabp3* mutant were almost as long as those of Col-0 (Figure 3a), and there was no significant difference in measured hypocotyl length (Figure 3c). However, under dark conditions, the hypocotyls of the *scabp3* mutant were significantly longer than those of Col-0 (Figure 3b, c). Furthermore, the PM H^+^-ATPase activity was significantly increased in the *scabp3* mutant. These results indicate that the increased PM H^+^-ATPase activity caused by *SCaBP3* deletion may promote hypocotyl elongation.

**Figure 3.**
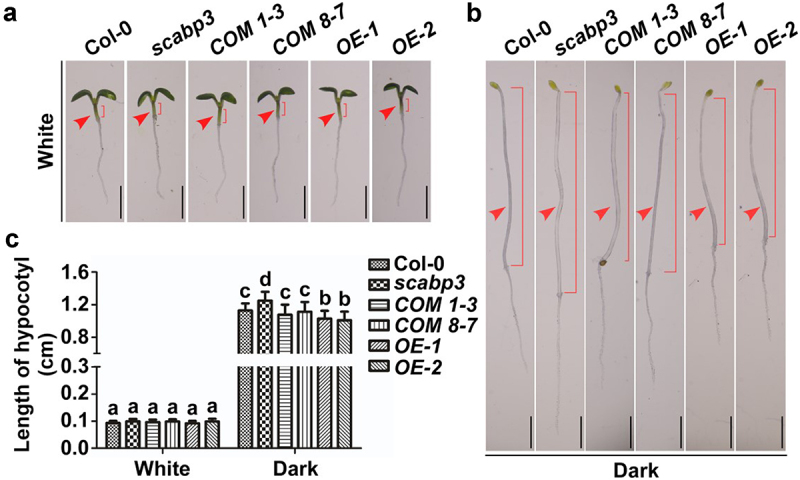
Hypocotyl growth phenotypes of Arabidopsis seedlings with different *SCaBP3* genotypes. (a) Hypocotyl growth phenotypes of Col-0, *scabp3* and its two complementation lines, and two *SCaBP3* overexpression lines under continuous white light conditions. (b) Hypocotyl growth phenotypes of Col-0, *scabp3* and its two complementation lines, and two *SCaBP3* overexpression lines under dark conditions. (c) Statistical analysis of the hypocotyl lengths in (a) and (b). Two-way ANOVA was used to determine statistical significance; significance differences between the experimental groups (P ≤ .05) are indicated by different lowercase letters. The red arrows indicate the hypocotyls, and the red brackets indicate the hypocotyl lengths.

These results are supported by the phenotypes of the complementation and overexpression lines. Under dark conditions, the hypocotyl elongation of the two complementation lines (COM 1–3 and COM 8–7) was almost indistinguishable from that of Col-0 but was significantly reduced in the two overexpression lines (OE-1 and OE-2) (Figure 3b, c). These results further indicate that changes in hypocotyl elongation in the *scabp3* mutant are caused by *SCaBP3* deletion and are closely related to changes in PM H^+^-ATPase activity. Decreased PM H^+^-ATPase activity may lead to inhibition of hypocotyl growth. In conclusion, SCaBP3-mediated regulation of PM H^+^-ATPase activity may be involved in the regulation of Arabidopsis hypocotyl elongation.

In this study, we found that SCaBP3 was able to interact with the C terminus of multiple members of the AHA family (AHA1/3/9/10) in yeast-two-hybrid assays. As an important proton transporter in plants, PM H^+^-ATPase is involved in almost every aspect of cellular life. Members of the AHA family have diverse physiological functions. For example, *AHA9* is expressed in anther tissues,^14^ and *AHA1* regulates the stomatal aperture in response to ABA signaling^13^ and participates in the control of hypocotyl elongation with AHA2.^10^ Therefore, the substrate diversity of SCaBP3 may be one of the reasons for its functional diversity, and we speculate that SCaBP3 may also play important roles in other physiological processes.

PM H^+^-ATPase provides the necessary energy for plant cell growth by establishing an electrochemical gradient across the cell membrane.^3^ At the same time, the regulation of H^+^-ATPase activity also plays an important role in plant hormone and stress responses.^3,15^ H^+^-ATPase activity is regulated by a variety of mechanisms in plants. Phosphorylation of serine/threonine residues in the autoinhibitory domain of H^+^-ATPase and binding of proteins such as 14-3-3 proteins are crucial for the regulation of the enzyme.^16–18^ The present study found that the Arabidopsis calcium-binding protein SCaBP3 is also an important negative regulator of H^+^-ATPase activity.

As the “master enzyme” of plant life processes, PM H^+^-ATPase plays important regulatory roles in growth, development, and stress responses. The genetic evidence in this study indicates that SCaBP3 is important for the regulation of PM H^+^-ATPase activity in plants.^11^ Because SCaBP3 interacts with a broad range of AHA family members, we speculate that SCaBP3-mediated regulation of H^+^-ATPase activity has diverse functions. Under dark conditions, the hypocotyl elongation rate of the *scabp3* mutant was significantly higher than that of the wild type (Col-0), suggesting that SCaBP3 may be involved in the regulation of hypocotyl elongation in Arabidopsis, but the underlying mechanism needs further study.

## Materials and methods

The Columbia (Col-0) ecotype of *Arabidopsis thaliana* was used in this study. The T-DNA insertion mutant *scabp3* was purchased from the Arabidopsis Biological Resource Center (ABRC) of the Ohio State University and identified as a homozygous mutant after screening. The transgenic plants used in this paper were produced by inoculating wild-type Col-0 and *scabp3* mutant plants with Agrobacterium GV3101 carrying the target plasmid. Positive seedlings were tested for the target protein, and T3 transgenic plants with stable inheritance were obtained.

Arabidopsis seeds were sterilized in 1 mL of seed disinfectant with inverting and shaking for 10 min. After the disinfectant was removed by aspiration, the seeds were washed with sterile ddH_2_O five to six times and then evenly sown on MS solid medium. The plates were sealed, placed at 4°C for 2–3 days and then placed in a continuous white light incubator. The petri dishes of the dark-treated experimental group were wrapped in dark foil and placed in the dark. After 4 days of growth at 22°C (CU-36L530; Percival Scientific Inc., Perry, IA, USA), the hypocotyl lengths of the seedlings were measured.

## Yeast two-hybrid assays

Three to five positive clones were selected, inoculated in 5 mL liquid yeast two- deficiency medium (SD-LW), and cultured at 28°C in a constant temperature shaker until the OD_600_ was about 1.0. Five hundred microliters of yeast cells was transferred to a 1.5-mL centrifuge tube and centrifuged for 1 min at 13,000 *g* at room temperature. The pellet was resuspended in 1 mL ddH_2_O, and the OD_600_ was measured. With 1 OD as the starting concentration, three serial 10-fold dilutions in of the yeast suspension were spotted onto four different media (SD-LW, SD-HLW, and SD-HLW + 1 mM 3-AT) and incubated at 28°C for 3–5 d before taking photos.

## Yeast RS-72 H^+^-ATPase complementation experiment

*S. cerevisiae* strain RS72 was used for complementation tests as described.^19^ SCaBP3 fragment was amplified and then the products were ligated into pMP1645 vector. Full-length AHA2, truncated AHA2, and point mutation of AHA2 were expressed under the control of the PMA1 promoter in the pMP1745 vector.^19^ All constructs were shown in Yang et al.^11^ Three to five positive clones were selected and inoculated into the liquid medium (SD-HL-glucose). The cultures were incubated in a 28°C shaker until OD_600_ was about 1.0. With OD_600_ = 0.4 as the starting concentration, and yeast growth (OD_600_ nm value) was measured every 30 min until 3 hours by using a microplate reader.^20^

## Accession numbers

Sequence data from this article can be found in the Arabidopsis Genome Initiative data libraries under the following accession numbers: AT4G26560 (SCaBP3), At4g30190 (AHA2), AT2G18960 (AHA1), AT5G57350 (AHA3), AT1G80660 (AHA9) and AT5G62670 (AHA11).
